# A Convenient Strategy for Studying Antibody Aggregation and Inhibition of Aggregation: Characterization and Simulation

**DOI:** 10.3390/pharmaceutics17040534

**Published:** 2025-04-19

**Authors:** Yibo Guo, Xi Chen, Guchen Fang, Xuejun Cao, Junfen Wan

**Affiliations:** State Key Laboratory of Bioreactor Engineering, Department of Bioengineering, East China University of Science and Technology, 130 Meilong Road, Shanghai 200237, China; y30200528@mail.ecust.edu.cn (Y.G.); chenxiscience@126.com (X.C.); y85240042@mail.ecust.edu.cn (G.F.); caoxj@ecust.edu.cn (X.C.)

**Keywords:** protein aggregation, MD simulation, bispecific antibody, single-chain fragment variable

## Abstract

**Background/Objectives:** Protein aggregation, particularly the aggregation of antibody-based drugs, has long been a significant challenge in downstream processes and formulation. While the inhibitory effects of excipients on aggregation have been extensively studied using early experimental characterization methods, complete formulation research requires significant amounts of antibodies and time, resulting in high research costs. **Methods:** This study proposed a quick and small-scale position-restrained simulation method which elucidated the mechanism of the reversible self-association (RSA) of antibodies and the influence of excipients on RSA under different conditions. We also validated the rationality of rapid and small-scale simulations through long-term (>1 μs) and large-scale (>1,000,000 atoms) simulations. **Results**: Through combing with simple stability characterization, the effects of different excipients on monomer residual content and the trend shown with concentration changes after thermal incubation were found to be similar to those observed in the simulations. Additionally, the formulation proposed by the simulations was validated using experimental characterization. **Conclusions**: Simulations and experiments revealed the mechanism and showed consistent trends, providing better understanding for aggregation research.

## 1. Introduction

In protein-based therapeutics, antibody drugs are often favored due to their high specificity and low immunogenicity in treating tumors and cardiovascular diseases [1]. These treatments typically require high doses to ensure therapeutic efficacy [2,3]. However, as the concentration of antibodies in the solution increases, various potential physical and chemical interactions within the antibody formulation can lead to instability and even the formation of new pyrogens [4,5].

The instability of antibodies, which are proteins, typically manifests as aggregation, including reversible and irreversible aggregation [6,7,8]. Reversible aggregation is generally mediated by weak interactions such as hydrophobic interactions, electrostatic interactions, and hydrogen bonds on the protein surface, while irreversible aggregation often results from chemical degradation and interactions between unfolded monomers, leading to the formation of high-molecular-weight aggregates. To inhibit these processes, scientists have conducted studies and found that adding certain excipients, including salts, amino acids, alcohols, sugars, and surfactants, to protein formulations is feasible [9,10,11,12,13,14]. Recent reports have also elucidated the mechanisms by which excipients inhibit protein aggregation. L-lysine and L-arginine, for instance, interact with proteins to increase steric hindrance and electrostatic repulsion, reducing the tendency of charged amino acids in proteins to form salt bridges, thereby inhibiting aggregation [15,16,17]. Glucose and sucrose affect protein hydration, reducing protein solvation entropy and increasing the enthalpic cost of aggregation, thereby delaying protein aggregation [18,19]. The addition of sorbitol, due to its hydrophilicity, results in preferential exclusion over its destabilizing effect on proteins [9].

The aggregation of specific proteins is influenced not only by their solvent environment but also by their intrinsic sequence and higher-order structures. Therefore, various theoretical calculation and prediction methods have been developed for predicting protein aggregation propensity, mainly including theoretical models for predicting aggregation-prone regions (APRs) and molecular dynamics (MD) simulations for atomic-level analysis [20,21,22,23]. In predicting APRs, Zyggregator integrates α-helix and β-sheet propensities, hydrophobicity, and charge effects based on native hydrogen exchange experiments [24]. AGGRESCAN predicts the aggregation propensity of exposed residues and generates structural ensembles through mutation comparisons to compute minimum energy structures [25]. Waltz deconstructs long amino acid sequences into multiple small hexapeptides, using polyalanine as a reference, to calculate the energy and score against experimental data [26]. Compared to the semi-empirical prediction methods mentioned above, MD simulations provide analyses with higher resolution for complex scenarios and have become an essential tool for investigating protein aggregation mechanisms, especially in early amyloid protein studies [27].

To study aggregation behavior, we chose a bispecific single-chain fragment variable (bsScFv) with high aggregation tendency (pI = 7.4) and chose succinic acid, Arg HCl, mannitol, and sucrose from different kinds of excipients (salt, amino acid, polyol, and sugar), as they are often used in excipients and are generally considered to inhibit aggregation via almost completely different modes of action [28]. We performed MD simulations in three parts, consisting of a pre-simulation at different initial positions, a simulation under different conditions of excipients, and a long-term, large-scale annealing simulation. An innovative position-restrained system was used in the simulations, significantly reducing the time required to obtain the expected simulation results. The three parts of the simulations were used to select representative aggregation areas, study the impact of different conditions on aggregation areas, verify the rationality of the first two simulations, and propose a possible formulation. Then, traditional experimental characterizations were conducted to validate the formulation. By integrating the results of the MD simulation with experimental characterization, we also investigated the aggregation inhibition mechanisms of different excipients and found a correlation between the simulation trajectories and experimental characterization results. This indicates that an appropriate simulation and simple characterization may reduce the cost of long-term stability experiments and help us to understand the interaction mechanism of different excipients.

## 2. Materials and Methods

### 2.1. Thermal Incubation Experiments

The sequences of the bsScFv were obtained from the WHO (https://www.who.int/teams/health-product-and-policy-standards/inn/inn-lists, accessed on 18 July 2023). The plasmids were synthesized by Qingke Biotechnology (Shanghai, China). BsScFv were expressed in our laboratory using HEK293f cells and purified using Eshmuno A (Merck, Darmstadt, Germany) and cation exchange chromatography (Nano-Micro, Suzhou, China) (Figure S1). This study involved four excipients: succinic acid, Arg·HCl, mannitol, and sucrose. Succinic acid and mannitol were purchased from Sigma-Aldrich (Shanghai, China). Sucrose and Arg·HCl were purchased from MACKLIN (Shanghai, China). All excipients were of purity grade (>99.8%). Proteins were incubated in EP tubes with each excipient at concentrations of 25, 50, 100, and 150 mM (with mannitol and sucrose additionally at 200 and 300 mM), a protein concentration of 10 mg/mL, and a total volume of 0.5 mL. The primary buffer was 10 mM Na_2_HPO_4_/NaH_2_PO_4_ at pH 6.0. Thermal incubation was conducted in Heratherm Compact Microbiological Incubators (Thermofisher, Waltham, MA, USA) at 40 °C for 7 days. All incubations were performed in triplicate. After incubation, samples were centrifuged (12,000 rpm, 5 min), and the supernatants were diluted ten times for measurements of UV absorption, SEC-HPLC, CD spectra, and DLS.

### 2.2. UV Absorption

UV absorption was used to determine the remaining soluble BsScFv content in the samples after thermal incubation. It was measured using an INESA-L6S (Yidian, Shanghai, China). Due to the excessively low absorbance of some samples at 280 nm, the absorbance of all samples was measured at 214 nm. The extinction coefficient of the target bsScFv at 280 nm was calculated to be 1.439 L/g in Expasy [29]. Pure BsScFv samples with concentrations of 0.05, 0.1, 0.25, 0.5, 0.75, and 1 mg/mL were prepared based on their absorbance at 280 nm. A standard curve was then plotted using their absorbance at 214 nm, which was utilized to determine the concentrations of the samples in the thermal incubation experiments. To exclude the absorption of excipients, the diluted samples were also exchanged into the main buffer using 30 kD ultrafiltration centrifuge tubes before measuring the UV absorbance.

### 2.3. SEC-HPLC

The remaining monomer rate was measured by SEC-HPLC (SHIMADZU CBM-10A VP PLUS, Kyoto, Japan). The column was Thermo Scientific MAbPac SEC-1 300×4 mm (Sunnyvale, CA, USA). The flow rate was set at 0.2 mL/min, the injection volume was set to 20 μL, and the mobile phase was 60 mM Na_2_HPO_4_/NaH_2_PO_4_ + 200 mM NaCl, pH 6.0. All samples were exchanged into the mobile phase buffer using 30 kD ultrafiltration centrifuge tubes before loading and then filtered through a 0.22 μm membrane.

### 2.4. Dynamic Light Scattering

The hydration diameter of proteins was measured using a Nano-ZS90 (Malvern Panalytical, Malvern, UK) through DLS in the range of 1 nm to 1.5 μm.

### 2.5. Circular Dichroism

CD spectra were measured using Chirascan (Applied Photophysics, Leatherhead, UK) with a bandwidth set to 1.5 nm and a time-per-point set to 1 s. Standard solutions with the same excipient concentrations as those in the diluted samples were prepared, and their CD spectra were measured to subtract the absorption contribution from the excipients.

### 2.6. Molecular Dynamics Simulations

MD simulations were performed using GROMACS (2023.3) [30]. Each simulation, except for the long-time annealing simulation, consisted of NVT (constant volume and temperature) and NPT (constant pressure and temperature) equilibrations, followed by an NPT production run. The three runs lasted 1, 10, and 300 ns, respectively. The integrator was md and the timestep was 2 fs. The constraint algorithm was LINCS and constraints were set to h-bonds. The thermostat was V-rescale, and the simulation temperature was set at 313 K to accelerate the molecular thermal motion, facilitating faster system equilibration without causing protein unfolding. The barostat was Berendsen in equilibration and Parrinello–Rahman in production, and the reference pressure and compressibility were set to 1.01325 bar and 4.5 × 10^−5^ bar^−1^, respectively.

The simulations were divided into three parts: a two-molecule simulation of bsScFv with different initial positions, a two-molecule simulation of bsScFv with different excipient concentrations, and a long-term annealing simulation. The box size for the first two parts of the simulation was 15 × 15 × 15 nm^3^ (containing 90,000–100,000 water molecules), while the box size for the third part of the simulation was 23 × 23 × 23 nm^3^ (containing 370,000–400,000 water molecules). The water model was TIP3P [31]. In the simulations with different initial positions, two bsScFv molecules were set in six different initial positions to study the aggregation characteristics of different surface regions of bsScFv. The shortest distance between the two bsScFv molecules was set to 1.6 ± 0.1 nm, and the shortest distance between one bsScFv and the mirror image of the other bsScFv was longer than 4.0 nm, enabling RSA to occur in the expected region. The displacement of bsScFv but not the excipients was restricted during NVT and NPT equilibrations, which allowed the excipients to sufficiently affect the aggregation of bsScFv during the pre-equilibrium. The short-range electrostatic potential cutoff and short-range van der Waals cutoff were set to 1.4 nm. Additionally, comm-mode and comm-grps were set to Angular and Protein, respectively, which helped to eliminate translational and rotational movements of the centroid of the two bsScFvs, making them more inclined to approach each other instead of escaping the simulation box. In two-molecule simulations with different excipient concentrations, a representative initial position from the initial position study was selected. Excipients were set at concentrations of 25, 50, 100, 150, and 300 mM. Long-term annealing simulations also consisted of two bsScFv molecules and the initial distance between the two molecules was set to be larger than 5 nm. The annealing settings were as follows: temperature first increased from 298 K to 363 K in 300 ns, was held at 363 K for 200 ns, then decreased from 363 K to 298 K in 300 ns, and finally was held at 298 K for 100 ns.

**Figure 1:**
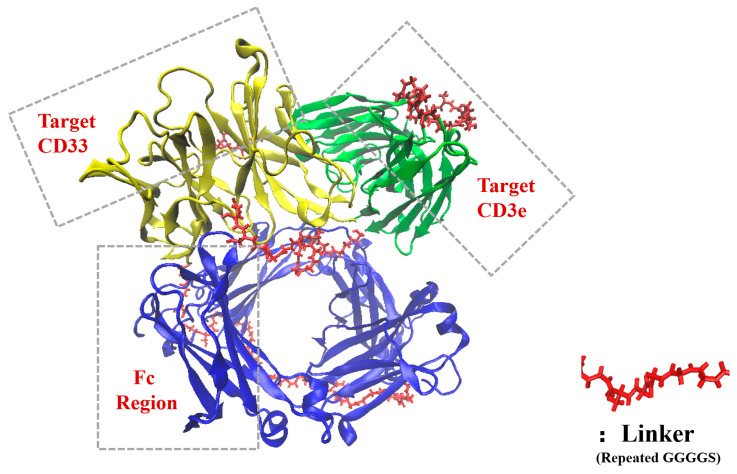
Optimized structure of bsScFv.

The structures of the two bsScFvs were initially constructed using the Alphafold2 casp14 model [32], and structures with a low pLDDT were adjusted to a flexible loop structure in Rosetta3 [33]. This adjustment was made for the convenience of folding in replica exchange MD (REMD). The fixed structures were then optimized in GROMACS using REMD with temperatures ranging from 308 K to 338 K with 20 replica, for a total duration of 1 μs [34]. The lowest-energy conformations after optimization are shown in Figure 1. The force field was Amber14sb [35], with the pH of the two bsScFv set to 6.0, and the pKa of each residue was calculated using H++4.0 (http://newbiophysics.cs.vt.edu/H++, accessed on 24 November 2023) [36,37,38].

The 3D structures of the four excipients were downloaded from the ATB database [39]. Structure optimization (B3LYP-D3/6-31G*) [40,41,42,43] and single-point energy (M062X-D3/def2TZVP) [44,45] were calculated in Gaussian16 [46], aiming to calculate the bond and angle parameters based on the Hessian matrix derived from frequency calculations and to determine the RESP2 charges [47] from the wavefunction obtained in single-point energy calculations. Electrostatic potential-colored van der Waals surface maps were plotted using VMD 1.9.3 [48] and Multiwfn 3.8 [49]. Multiwfn was also used to calculate the RESP2 charges of the excipients; the charge distribution was set to 50% for both the vacuum and implicit solvent models (the implicit solvent model used was SMD, with water as a solvent). Topological files for the excipients were generated using Sobtop (Tian Lu, Sobtop, Version 1.0 dev5, http://sobereva.com/soft/Sobtop, accessed on 2 July 2025), where bond and angle parameters were generated from the Hessian matrix and dihedra land improper parameters were from GAFF [50].

Gmx_MMPBSA is a powerful tool frequently used to calculate binding energy related to proteins [51]. In this study, gmx_MMPBSA1.6.3 was used to calculate the aggregation binding energy of bsScFv and the binding energy between bsScFv and excipients. Binding energy was decomposed into van der Waals interaction energy, electrostatic interaction energy, Poisson–Boltzmann solvation energy, and the contribution of each residue.

### 2.7. Experimental and Simulated Preferential Interaction Coefficient

The experimental preferential interaction coefficient (Γ_23_) was measured using a Wescor VAPRO 5600 vapor pressure osmometer (Wescor, Logan, UT, USA). Γ_23_ for bsScFv with each excipient was measured at concentrations in the range of 0−0.3 M, according to [52]. Samples with 10 mM Na_2_HPO_4_/NaH_2_PO_4_ at pH 6.0 and 10 mg/mL bsScFv were prepared as binary series of solutions. Samples with 10 mM Na_2_HPO_4_/NaH_2_PO_4_, 20 mg/mL bsScFv, and 0−0.3 M excipients were prepared as ternary series of solutions. Each measurement was conducted in triplicate.

The simulated Γ_23_ was calculated according to [53]:

$$\Gamma_{23}\left(r,t\right)=n_{3}\left(r,t\right)-n_{1}\left(r,t\right)(\frac{n_{3}-n_{3}\left(r,t\right)}{n_{1}-n_{1}\left(r,t\right)})$$

where *n*_1_ and *n*_3_ represent the total number of water and excipients in the simulation system, and *n_i_*(*r*,*t*) represents the number of *n_i_* molecules with the shortest distance *r* from the protein at time *t*. *r* here was usually set to 8 Å.

## 3. Results and Discussion

### 3.1. Two-Molecule Simulations with Different Initial Positions

**Figure 2:**
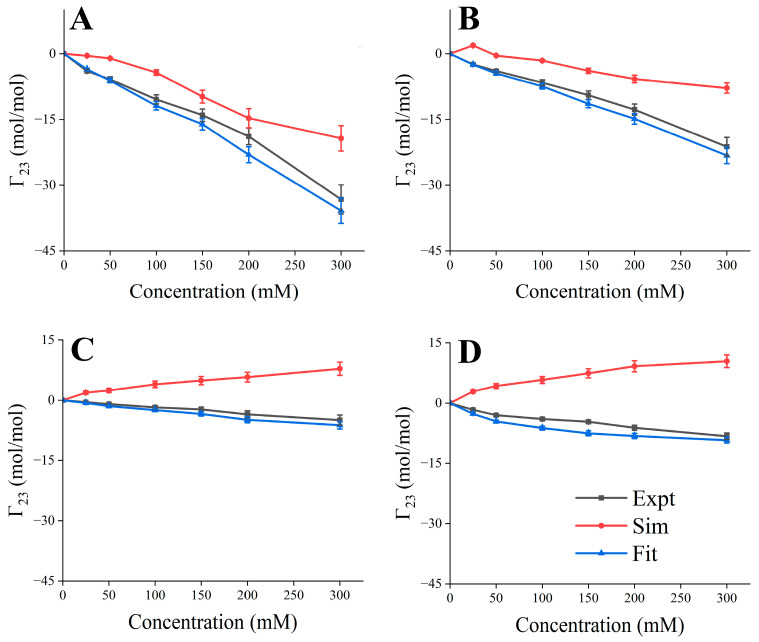
Comparison of experimental and simulated Γ_23_ as a function of excipient concentration. (**A**) Succinic acid; (**B**) Arg·HCl; (**C**) mannitol; (**D**) sucrose.

The existing force field often cannot accurately reflect the interaction among excipients, proteins, and water; for example, sugar/alcohol will produce a large amount of self-aggregation and additional protein binding [54,55]. In order to study the inhibitory effect of the concentration of excipients on the formation of RSA, the parameters of the LJ potential of the excipient topology file were fitted to better describe the interactions between solutes (Table S1). A 300 ns simulation with both fitted and unfitted LJ parameters for each condition was performed, and the last frame trajectory was used to calculate Γ_23_, and Γ_23_ with fitted parameters, showing high consistency with the values observed in the experiments (Figure 2). All subsequent simulations used the modified LJ parameters.

**Figure 3:**
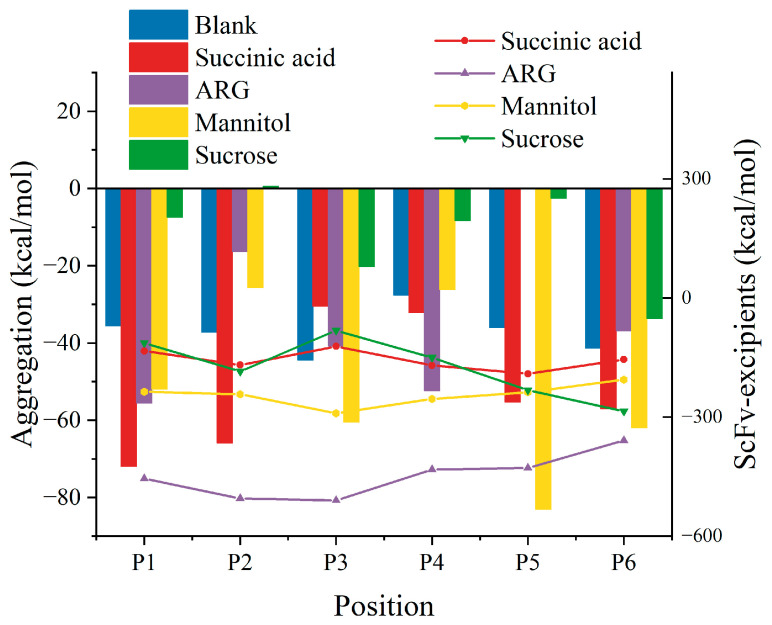
Binding energy of bsScFv aggregates from different initial positions (bar chart) and binding energy between bsScFv and each excipient (point-line graph).

Since analyzing all-atom molecular dynamics requires very high computing power, and the formation of aggregated nuclei is not easily reflected in microsecond timeframes, simulations are mainly used to study the mechanism of RSA formation. Due to the flexible structure in bsScFvs, which usually possess significant hydrophobicity to ensure antigen–antibody complementarity, these regions are also prone to forming transient complexes due to hydrophobic interactions. Considering this, we constructed six systems containing two bsScFv molecules with different initial positions (Figure S1) to cover APRs of bsScFv as comprehensively as possible during the simulations. Four excipients were added to each system to study their inhibitory effects on bsScFv RSA. Concentrations of excipients were set at levels commonly used in protein drug formulations (25 mM succinic acid, 150 mM ARG+, 150 mM mannitol, and 300 mM sucrose). Notably, the binding energy between antibodies and the inhibitory effects of excipients on bsScFv RSA varied significantly among different systems (Figure 3). Sucrose showed the best inhibitory effect on RSA at all positions, suggesting the comprehensive shielding of potentially aggregating residues on the bsScFv surface. ARG+ demonstrated inhibitory effects at some initial positions (P2, P3, P5, P6), even causing repulsion between two bsScFv molecules at P5. In contrast, succinic acid and mannitol tended to enhance bsScFv RSA at most initial positions, and their binding energy with bsScFv was minimal.

The decomposed binding energy results (Figure S3) revealed that electrostatic interactions were the main contributors to bsScFv RSA. Both succinic acid and ARG+, due to their net surface charges, exhibited binding energy with bsScFv that was predominantly from electrostatic interactions. Mannitol, despite lacking enriched surface charges, still showed stronger electrostatic interactions than van der Waals forces, with its six hydroxyl groups offering numerous hydrogen bond donors and acceptors with bsScFv. Under the influence of sucrose, the number of hydrogen bonds between oligomers was the lowest, indicating that sucrose effectively blocked the hydrogen bond-forming sites on bsScFv, reducing the impact of hydrogen bonds on RSA.

The RSA sites (Figure S4h) were mainly concentrated in the highly flexible loops of the two CDRs, the linker, and the α-helix at the C-terminus of the constant region. In particular, RSA regions in P1, P3, and P5 (Figure S4a,c,e) were primarily located in the long linker connecting the constant region (containing six repeats of GGGGS) and the Fc region. In contrast, RSA regions in P2, P4, and P6 (Figure S4b,d,f) were dispersed in CDR1, CDR2, and the nearby linker. Flexible structures have a higher probability of RSA, with stable interaction distances mostly ranging between 0.15 and 0.2 nm.

We further analyzed all binding sites between bsScFv and the excipients, focusing on residues with significant binding energy contributions (<−5 kcal/mol) (Figure S5). Succinic acid and ARG+ primarily bound to residues with opposite charges (Figure S5c,f). However, trajectory analysis revealed that succinic acid appeared infrequently in RSA regions (Figure S6A), mostly remaining free in solution and not effectively shielding the interactions between charged residues within RSA regions. ARG+, in contrast, frequently appeared in RSA regions (Figure S6B), exhibiting a shielding effect on GLU and ASP residues, thereby reducing the space for electrostatic interactions between these residues and lowering their RSA binding energy contributions. Although mannitol also appeared infrequently in RSA regions (Figure S6C), it interestingly tended to distribute within the interior of bsScFv, interacting with multiple residues (Figure S6(C1,C2)). This characteristic could also cause mannitol to act as a bridge connecting similarly charged residues between aggregates (Figure S6(C5,C6)), thereby accelerating the formation of reversible oligomers from the native conformation. The RSA area was the smallest in the sucrose-added groups, regardless of the initial position (Figure S3c). Sucrose oligomers occupied most of the contact space between antibodies, thereby hindering further interactions between protein molecules. Although sucrose interacted with various residues in the antibody, its overall binding energy was weak (Figure S5k), implying that sucrose might temporarily lose its shielding effect at these weak binding sites due to some intense thermal motion.

### 3.2. Excipient Concentration Simulation

**Figure 4:**
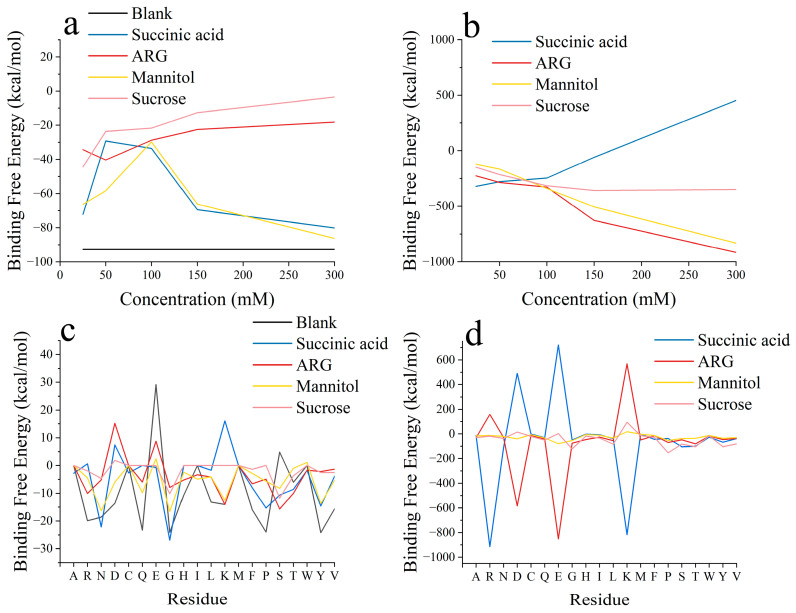
RSA binding energy, excipient–bsScFv binding energy, and corresponding residue decomposition. (**a**) RSA binding energy of bsScFv; (**b**) binding energy between bsScFv and excipients; (**c**) RSA binding energy contributions of each residue; (**d**) binding energy between each residue and excipients.

**Figure 5:**
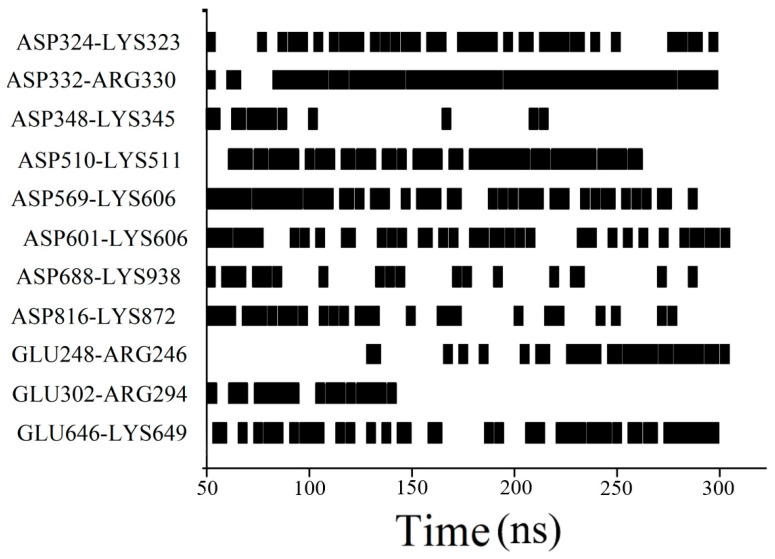
Significant salt bridge changes in the Arg·HCl group.

Based on the above study performed with different initial positions, we further investigated the effect of excipient concentration on RSA. P6, which has the highest RSA formation energy, was chosen as the initial position. Succinic acid at a concentration of 50 mM had a much better inhibitory effect on RSA than that at 150 mM (Figure 4). This does not mean that 150 mM succinic acid has a higher inhibitory effect on RSA formation than 50 mM succinic acid, but rather, that the inhibitory effect on RSA at a concentration of 50 mM is close to saturation. Despite the initial positions being set to probably aggregate at specified sites, in the 150 mM succinic acid system, the two bsScFv molecules still deviated from each other and interacted with the mirror image of one another (Figure S6). Differing from succinic acid, the increase in ARG+ concentration was more favorable for ARG+ in competing for the negatively charged regions on the bsScFv surface, and ARG+ primarily bound to the residues GLU and ASP to reduce the RSA driving force provided by electrostatic interactions. We also found that ARG+ affected the formation and breaking of salt bridges between some residues in bsScFv (Figure 5), while other excipients did not have such an obvious effect on salt bridges, meaning that ARG+ may change the conformation of bsScFv. Significant self-association of ARG+ was observed in the system (Figure S8), which was not observed at high concentrations of succinic acid. This association weakened the electrostatic shielding effect of high-concentration ARG+.

As a good hydrogen bond donor, mannitol could interact with most residues containing hydrogen bond acceptor oxygens. Consequently, the binding energy between mannitol and residues including ASP, GLN, GLU, PRO, SER, and THR was higher than that with other residues (Figure 4d). Its densely distributed surface electrostatic charges also enhanced its interactions with the residues ASP and GLU. In the blank control of the RSA region, the residues GLU and SER exhibited repulsive effects on RSA formation (Figure 4a). However, in the presence of mannitol, their repulsive effects transformed into RSA-enhancing effects, while the binding energy contributions from other hydrogen-bonds residues decreased. Mannitol bound to the RSA region via hydrogen bonds and acted as a bridge between two bsScFv molecules.

Sucrose had the most significant inhibitory effect on RSA formation (Figure 4a). When the sucrose concentration reached 300 mM, the two bsScFv molecules were unable to approach each other due to the steric effect provided by sucrose, with the shortest distance exceeding 7 Å (Figure S9D). Consequently, the binding energy between the two bsScFv molecules dropped to zero (Figure 4a). The growth in binding energy between sucrose and bsScFv became nearly flat, indicating that sucrose had almost completely blocked all binding residues on the bsScFv surface at this concentration. The binding energy between sucrose and various residues was significantly lower than that of other excipients (Figure 4d), but sucrose had a stronger effect on reducing the RSA binding energy provided by these residues. The binding energy contributions of all residues were less than −5 kcal/mol, except for the residues GLY and SER, which were part of the linker region in bsScFv and had a highly flexible random coil structure. When sucrose attempts to bind to the residues GLY or SER, the active molecular thermal motion in this region may disrupt the shielding effect of sucrose, thereby weakening its overall blocking effect.

The inhibitory effect of each excipient on the RSA in the simulation and the monomer loss in the thermal incubation experiment showed good consistency with the concentration change, especially for succinic acid and sucrose, and the saturation concentrations corresponding to their inhibitory effects were consistent in the simulation and the experiment. Based on the properties of these excipients, unique inhibitory mechanisms on bsScFv RSA can be proposed. Succinic acid, a common buffer component, binds infrequently to the positively charged residues on the surface or interior of bsScFv. Contrary to our traditional understanding of electrostatic interactions, succinic acid does not accumulate near ARG or LYS residues despite its high binding energy with these residues. By distributing evenly in the solution, succinic acid creates an electrostatic environment that repels areas of high negative charge density on the bsScFv surface (Figure S7). However, the bsScFv surface also contains regions of strong hydrophobic interactions, leading to RSA formation when only succinic acid is present. In contrast, ARG+, another excipient with a net charge, primarily interacts with bsScFv through electrostatic interactions, followed by hydrogen bonds. This binding covers a much greater extent than succinic acid and shields the electrostatic interactions on aggregation-prone regions (APRs). Increasing ARG+ enhances the binding energy and competes for electrostatic interaction sites within the RSA structure, thereby inhibiting or disrupting RSA. Mannitol has a weak effect in inhibiting RSA formation. Although mannitol has a very high binding energy with bsScFv, it binds to various surface residues in various ways and lacks effective steric hindrance. Sucrose plays a dominant role in inhibiting RSA, as evidenced by the fact that more than 80% of soluble proteins and 40% of monomers remained after seven days of thermal incubation with bsScFv when sucrose was present in the formulation. By forming large oligomers on the bsScFv surface, sucrose provides an extremely high steric effect.

### 3.3. Long-Time Annealing Simulation

**Figure 6:**
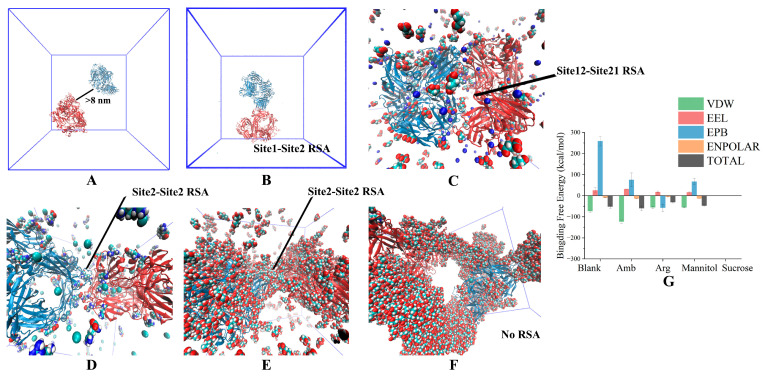
The structure of RSA simulation using the long-term annealing method in a large system. (**A**) Original structure; (**B**) RSA in blank; (**C**) RSA in 50 mM succinic acid; (**D**) RSA in 50 mM Arg·HCl; (**E**) RSA in 300 mM mannitol; (**F**) RSA in 300 mM Sucrose; (**G**) Formation energy of RSA.

We performed several long-term annealing simulations (1.1 μs) at a fixed excipient concentration with two bsScFv molecules in a 23 × 23 × 23 nm^3^ box. The addition of excipients was as same as for CD characterization, and each condition was repeated three times with different initial velocities. Compared with the short-term constant temperature simulation, long-term annealing can allow the protein structure to traverse multiple minimum-energy structures. In these simulations, we also discovered multiple ways in which bsScFv forms RSA under different conditions (Figure 6). In these large systems, the initial distance of two bsScFv was great enough. Theoretically, each APR of bsScFv had the same potential to promote the formation of RSA. Under this premise, succinic acid and mannitol exhibited weak inhibitory effects on the formation of RSA, while Arg·HCl and sucrose demonstrated better inhibitory effects. Consistent with the simulation results in the previous section, after the system was expanded, sucrose still exhibited a strong steric hindrance effect. Although mannitol covered a large area on the surface of bsScFv, its effect on reducing the binding energy for the formation of RSA was not obvious. Overall, we verified the feasibility of using small systems and short-time simulations to study RSA formation instead of large systems and long-term simulations.

Based on the interaction mechanism and trend between excipients and bsScFv, we conducted corresponding acceleration experiments and characterization to verify the accuracy of the simulation. We also proposed a possible formulation based on RSA formation energy and conducted experimental verification (50 mM succinic acid + 150 mM Arg·HCl + 200 mM Sucrose). The choice for the predicted formulation was based on the concentration at which the inhibitory effect provided by each formulation molecule approached saturation.

### 3.4. Soluble BsScFv Characterization

**Figure 7:**
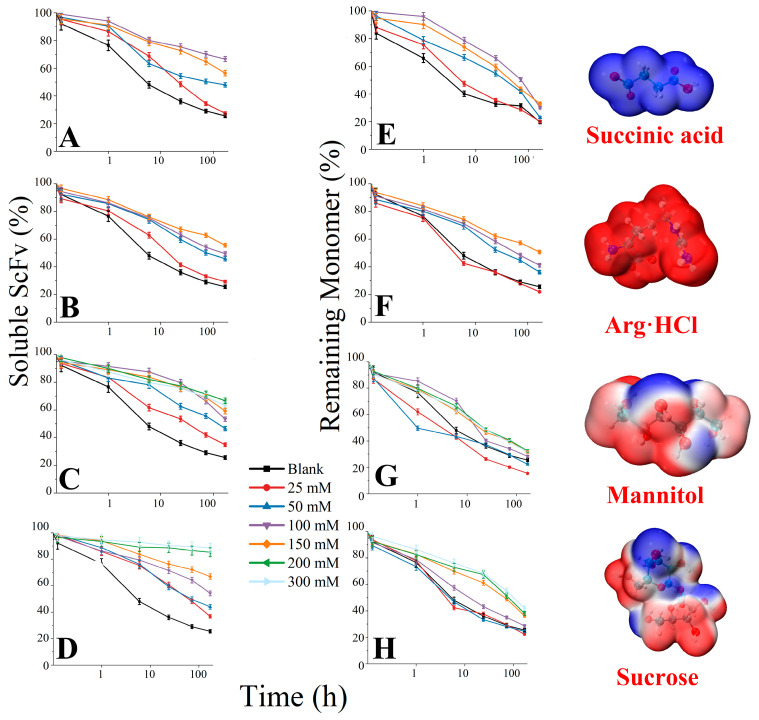
The remaining soluble bsScFv and monomer during 7-day thermal incubation. (**A**–**D**) Remaining soluble bsScFv in succinic acid, Arg·HCl, mannitol, and sucrose, respectively; (**E**–**H**) Remaining monomer rate in succinic acid, Arg·HCl, mannitol, and sucrose, respectively.

We centrifuged the samples after 7 days of thermal incubation (12,000 rpm, 5 min) to remove insoluble precipitates, then diluted them ten times and measured UV absorbance at 214 nm to characterize the inhibitory effects of excipients on the formation of soluble and insoluble aggregates (Figure 7). Samples with only the primary buffer were used as the blank. When the succinic acid concentration exceeded 100 mM (Figure 7A), the soluble residual content decreased and the increase in succinic acid concentration had a significant inhibitory effect on the conversion of monomers to aggregates in the early stage of thermal incubation; however, this improvement was not significant compared to the blank in the later stage of thermal incubation (Figure 7E). This indicated that there is an optimal concentration of succinic acid for aggregation inhibition. As the concentration of Arg·HCl increased, its effects on inhibiting aggregation were significantly improved (Figure 7B,F). Among the five excipients, Arg·HCl also had the best inhibitory effect on the conversion of monomers to oligomers (the monomer residual content was 50.67% on the 7th day). The inhibitory effect of mannitol on the formation of aggregation reached saturation at 100 mM, and the soluble residual content was still 60–70% after 7 days (Figure 7C), while the inhibitory effect on the formation of oligomers was not obvious and even promoted the formation of oligomers at lower concentrations (Figure 7G). High concentrations of sucrose have a significant effect on inhibiting the formation of irreversible aggregation and oligomers (Figure 7D,H). On the seventh day, the soluble residual content in 300 mM sucrose was still as high as 88.66%, and the remaining monomer rate also reached 42.02%. The trend of the inhibitory effect of the four excipients on aggregation with concentration changes was the same as their RSA inhibition trend in the simulation.

**Figure 8:**
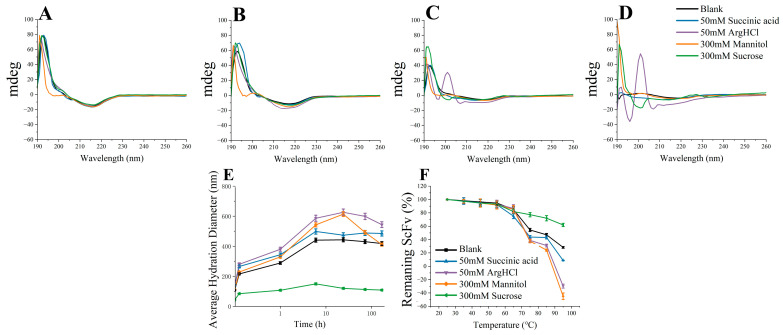
Characterization of CD spectra, hydration diameter, and thermal stability: (**A**) 0d CD spectra; (**B**) 1d CD spectra; (**C**) 3d CD spectra; (**D**) 7d CD spectra; (**E**) average hydration diameter; (**F**) thermal stability.

The characterization of the bsScFv structural stability was performed at a fixed excipient concentration (Figure 8). Between 3d and 7d, the increase in random coil structures observed in the Arg·HCl group indicated that the residues that provided weak interactions gradually dissociated under the effect of Arg·HCl (Figure 8C,D), which was also indicated by simulations. From the hydrated diameter curves of different excipients (Figure 8E), it could be seen that sucrose had a significant effect on maintaining the growth of oligomers. The hydration diameter of the Arg·HCl group was always higher than that of the other groups and reached more than 600 nm on the third day, indicating that the growth rate of oligomers in the Arg·HCl group was fast. Additionally, the mannitol group had a higher average hydration diameter during the thermal incubation process, and the rate of decrease in the average hydration diameter in the later stages of thermal incubation was much higher than that of the other groups. Combining the results of the residual soluble and residual monomer, it can be concluded that mannitol promoted the rapid increase in the average hydration diameter by facilitating the conversion of monomers to oligomers during the early stage of thermal incubation. However, once the monomer residual content dropped to a certain level, the effect of mannitol on promoting monomer aggregation weakened, but it still maintained the ability to promote the transition of soluble aggregation nuclei into insoluble precipitates.

The thermal stability was characterized by the soluble residual content after thermal incubation at different temperatures for 10 min (Figure 8F). The soluble residual content was also determined by means of UV absorbance at 214 nm, but buffers of these samples were not replaced with ultrafiltration centrifuge tubes. Similarly, sucrose had a higher inhibitory effect on the growth of oligomers at high temperatures than other excipients. The addition of succinic acid slightly reduced the thermal stability. The soluble residual content of mannitol and Arg·HCl groups dropped to a negative number because the absorption value of the buffer needed to be subtracted when calculating the residual content. The absorption value of the sample after 95 °C bath for 10 min was lower than the absorption value of the buffer, which indicated that mannitol and Arg·HCl were largely involved in the formation of aggregation and precipitate with the aggregates. The simulation results also indicated that the binding energy between mannitol and bsScFv was the strongest, and it was favorable for the formation of RSA, which is possibly the reason why the monomers in the mannitol group were more likely to convert into oligomers.

### 3.5. Validation of Predicted Formulation

**Figure 9:**
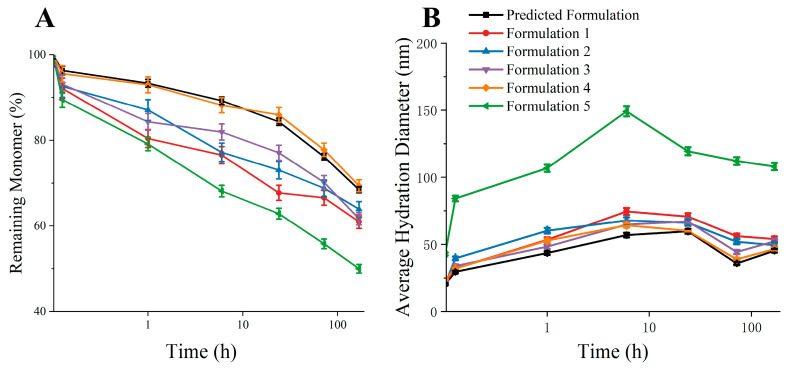
Thermal incubation experiment results of multiple formulations. (**A**) Remaining monomer after thermal incubation experiment; (**B**) average hydration diameter after thermal incubation experiment.

We conducted thermal incubation experiments under multiple formulations to verify the feasibility of the predicted formulation (Table 1). From the results of the thermal incubation experiment (Figure 9), it could be seen that the aggregation inhibition effect of the predicted formula was comparable to that of Formula 4 with a high concentration of excipients, which led the residual monomer rate to be close to 70% at 7d. The aggregation inhibition effect of Formulations 1, 2, and 3 was not as good as the predicted formulation. Compared with Formulation 5, it can be seen that mannitol had a promoting effect on the aggregation of bsScFv. The above results indicate that it was feasible to predict the optimal formula for aggregation inhibition through simulation.

## 4. Conclusions

Protein aggregation has long been recognized as a persistent and complex process. This study characterized and simulated the aggregation phenomenon, revealing the inhibitory effect and mechanisms of bsScFv aggregation. Additionally, it used innovative simulation methods and explored the commonalities between large systems and small systems, proposing the feasibility of using simple simulations as a starting point for aggregation experiments. We also found that GLY and SER in the linker contributed significantly to the formation of RSA, and the excipient provided inhibitory effects on the aggregation of linker contributions. We will further investigate the differences in aggregation mechanisms between bsScFv containing a linker and normal monoclonal antibodies in the future. Overall, MD simulations have extremely high potential to reduce the cost and time associated with traditional long-term stability experiments, facilitating the study of other excipient inhibitory mechanisms and the rapid screening of protein formulations.

## Figures and Tables

**Table 1:** Multiple formulations for validating the predicted feasibility of the predicted formulation.

Name	Formulation
Predicted Formulation	50 mM succinic acid + 150 mM Arg·HCl + 200 mM Sucrose
Formulation 1	15 mM succinic acid + 50 mM Arg·HCl + 50 mM Sucrose
Formulation 2	30 mM succinic acid + 100 mM Arg·HCl + 100 mM Sucrose
Formulation 3	75 mM succinic acid + 200 mM Arg·HCl + 150 mM Sucrose
Formulation 4	100 mM succinic acid + 300 mM Arg·HCl + 300 mM Sucrose
Formulation 5	50 mM succinic acid + 150 mM Arg·HCl + 200 mM Sucrose + 100 mM Mannitol

## Data Availability

The raw data supporting the conclusions of this article will be made available by the authors on request. The data are not publicly available due to the too large original files.

## Supplementary Materials

The following supporting information can be downloaded at: https://www.mdpi.com/article/10.3390/pharmaceutics17040534/s1, Figure S1. Expression and purification information of bis-ScFv characterized by SDS-page and SEC-HPLC. Figure S2. Six different initial positions of two-molecule simulations. Figure S3. Binding free energy and aggregation contact area in two-molecule simulations under different initial position conditions. Figure S4. Aggregation sites of bis-ScFv under different initial position conditions. Figure S5. Binding sites between excipients and bis-ScFv. Figure S6. Aggregation structures under different initial positions and excipients conditions. Figure S7. Aggregation trajectories of two bis-ScFv in 50 mM and 150 mM succinic acid. Figure S8. Self-aggregation of ARG+ in 150 mM ARG+ system. Figure S9. Contact structures between excipients and bis-ScFv at different excipient concentrations. Table S1. Modified LJ parameters of four excipients.
