# Supplementary material for: A Convenient Strategy for Studying Antibody Aggregation and Inhibition of Aggregation: Characterization and Simulation
Source: Pharmaceutics. 2025 Apr 19;17(4):534. doi: 10.3390/pharmaceutics17040534 (PMC12030238; doi:10.3390/pharmaceutics17040534)
Supplement: Supplementary file 1 [file pharmaceutics-17-00534-s001.zip › pharmaceutics-3571960-supplementary.pdf]

SUPPLEMENTARY INFORMATION

A Convenient Strategy for Studying Antibody Aggregation and Inhibition of Aggregation: Characterization and Simulation

Yibo Guo[1], Xi Chen[1], Guchen Fang[1], Xuejun Cao[1], Junfen Wan[1]

[1] State Key Laboratory of Bioreactor Engineering, Department of Bioengineering, East China University of Science and Technology, 130 Meilong Road, Shanghai, 200237 China

Corresponding author:

Junfen Wan

Email: wanjunfen@ecust.edu.cn

Figure S1. Expression and purification information of bis-ScFv characterized by SDS-page and SEC-HPLC.

Figure S2. Six different initial positions of two-molecule simulations.

Figure S3. Binding free energy and aggregation contact area in two-molecule simulations under different initial position conditions.

Figure S4. Aggregation sites of bis-ScFv under different initial position conditions.

Figure S5. Binding sites between excipients and bis-ScFv.

Figure S6. Aggregation structures under different initial positions and excipients conditions.

Figure S7. Aggregation trajectories of two bis-ScFv in 50 mM and 150 mM succinic acid.

Figure S8. Self-aggregation of ARG+ in 150 mM ARG+ system.

Figure S9. Contact structures between excipients and bis-ScFv at different excipient concentrations.

Table S1. Modified LJ parameters of four excipients.

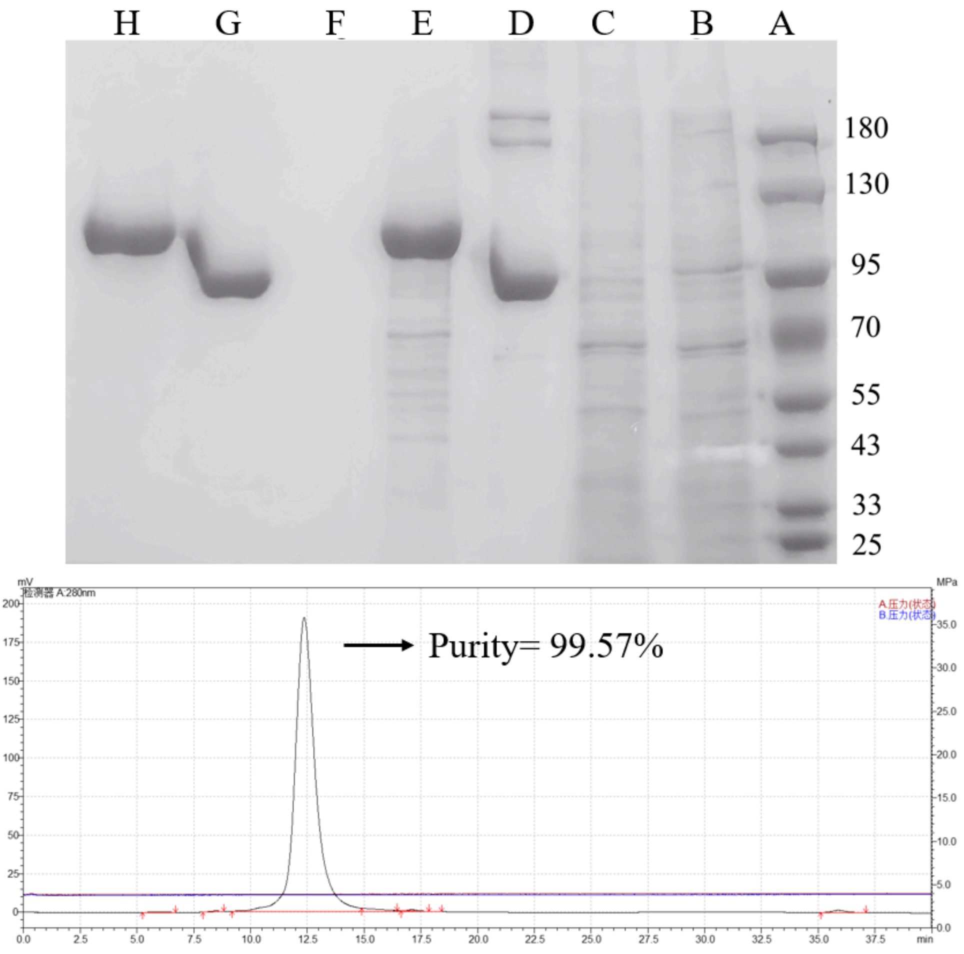

Figure S1. Expression and purification information of bis-ScFv characterized by SDS-PAGE and SEC-HPLC. (A) Marker. (B) Cell supernatant. (C) Protein A chromatography flow-through. (D) Protein A chromatography elution (non-reducing). (E) Protein A chromatography elution (reducing). (F) CEX chromatography flow-through. (G) CEX chromatography elution (non-reducing). (H) CEX chromatography elution (reducing). Sample after CEX chromatography was used to determine purity in SEC-HPLC.

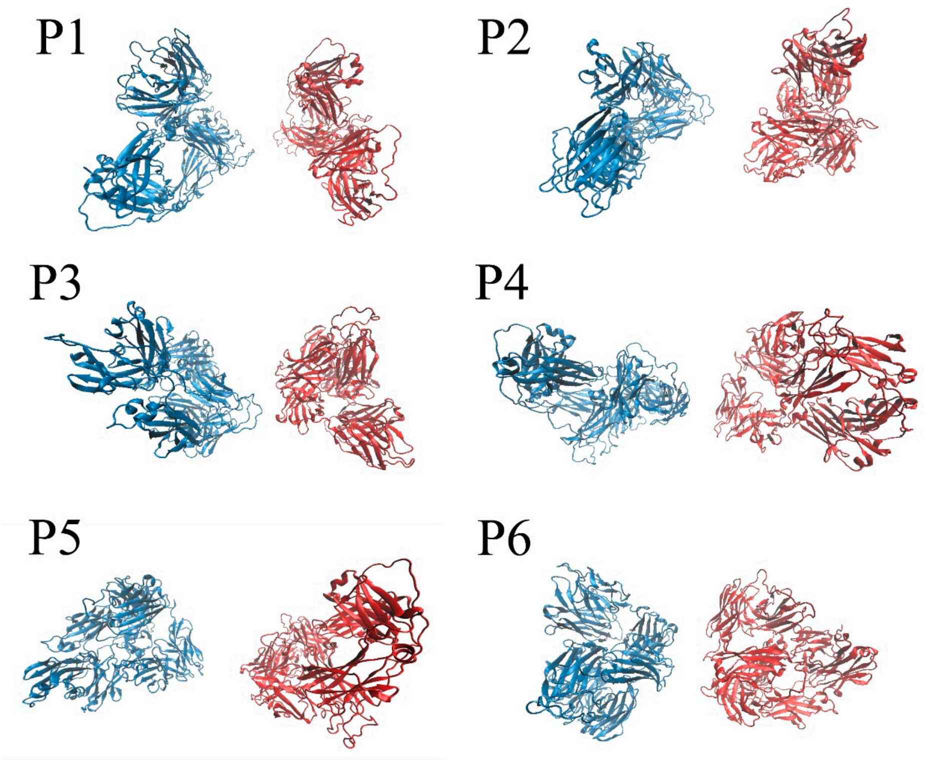

Figure S2. Six different initial positions of two-molecule simulations. These structures were obtained by manual adjustments in VMD, ensuring that the initial minimal distance between the two bis-ScFv structures is 1.6 ± 0.1 nm.

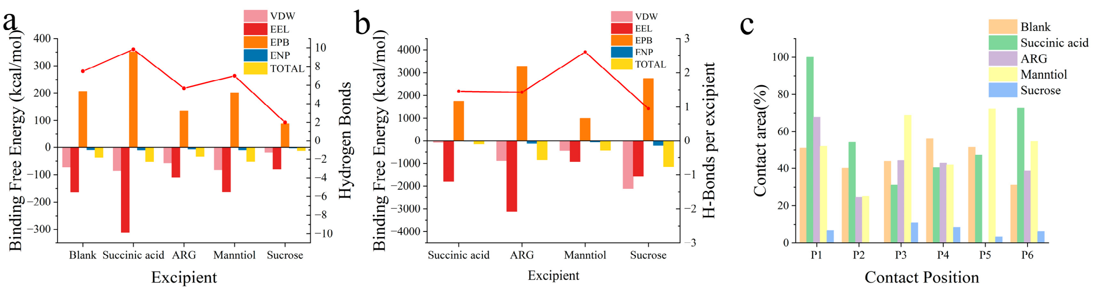

Figure S3. Binding free energy and aggregation contact area in two-molecule simulations under different initial position conditions. (a) Aggregation free energy; (b) Binding free energy of excipient-bis-ScFv; (c) Contact area between two bis-ScFv in the aggregated structure. The contact area was determined by calculating the number of atom pairs with a distance of less than 0.3 nm between the two bis-ScFv structures in the trajectory. The contact area was then normalized to 100% based on the contact area of the P1 + succinic acid structure, which had the maximum contact area.

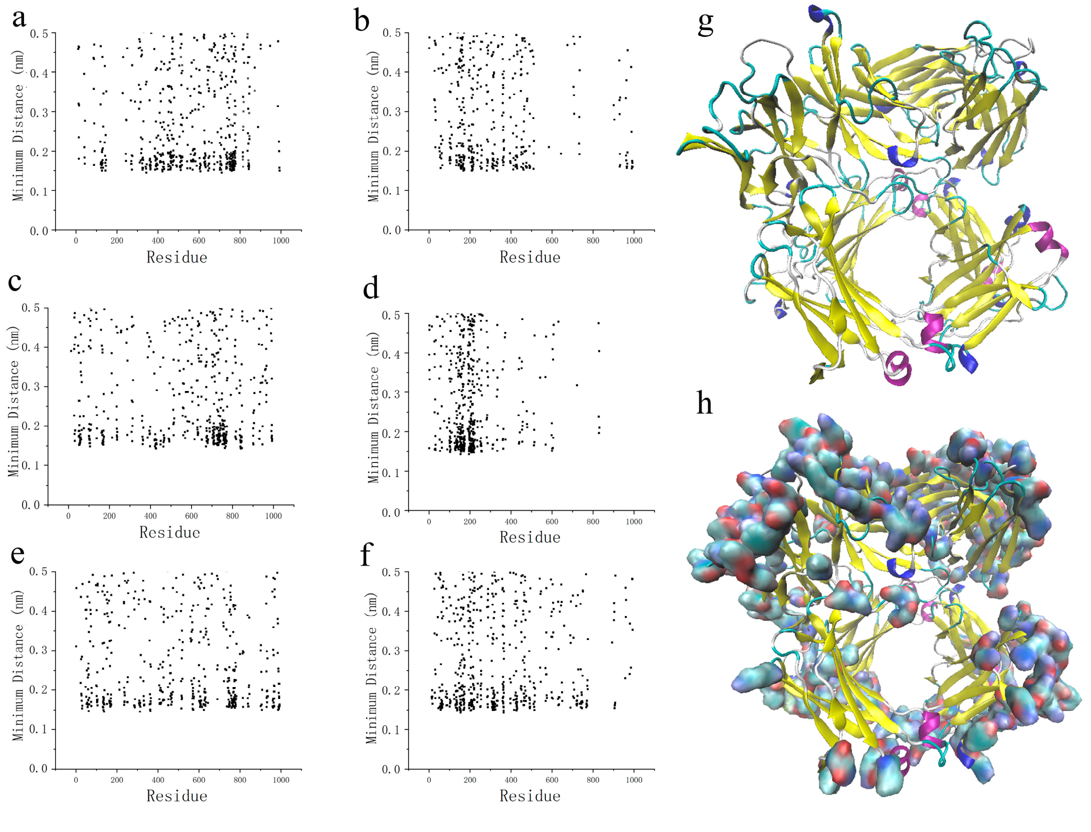

Figure S4. Aggregation sites of bis-ScFv under different initial position conditions. (a-f) The aggregation positions of P1-P6, respectively. Each data point represents the number of occurrences and the distance of the corresponding excipient near the residues. Denser data points indicate more frequent residue-excipient interactions. (g) Structure of bis-ScFv. (h) Location of bis-ScFv aggregation sites in the structure.

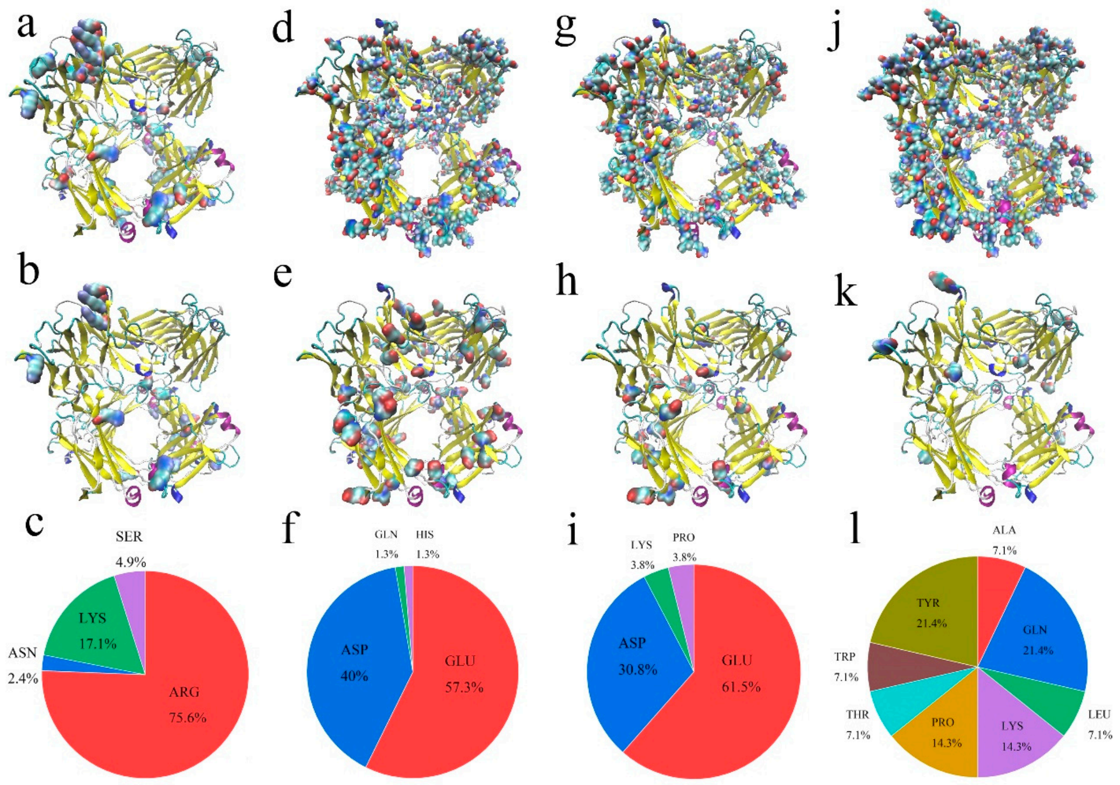

Figure S5. Binding sites between excipients and bis-ScFv. (a-c) The binding sites of succinic acid to bis-ScFv, the sites with strong binding energy and the ratio of binding residues. (d-f) The binding sites of ARG to bis-ScFv, the sites with strong binding energy and the ratio of binding residues. (g-i) The binding sites of mannitol to bis-ScFv, the sites with strong binding energy and the ratio of binding residues. (j-l) The binding sites of sucrose to bis-ScFv, the sites with strong binding energy and the ratio of binding residues.

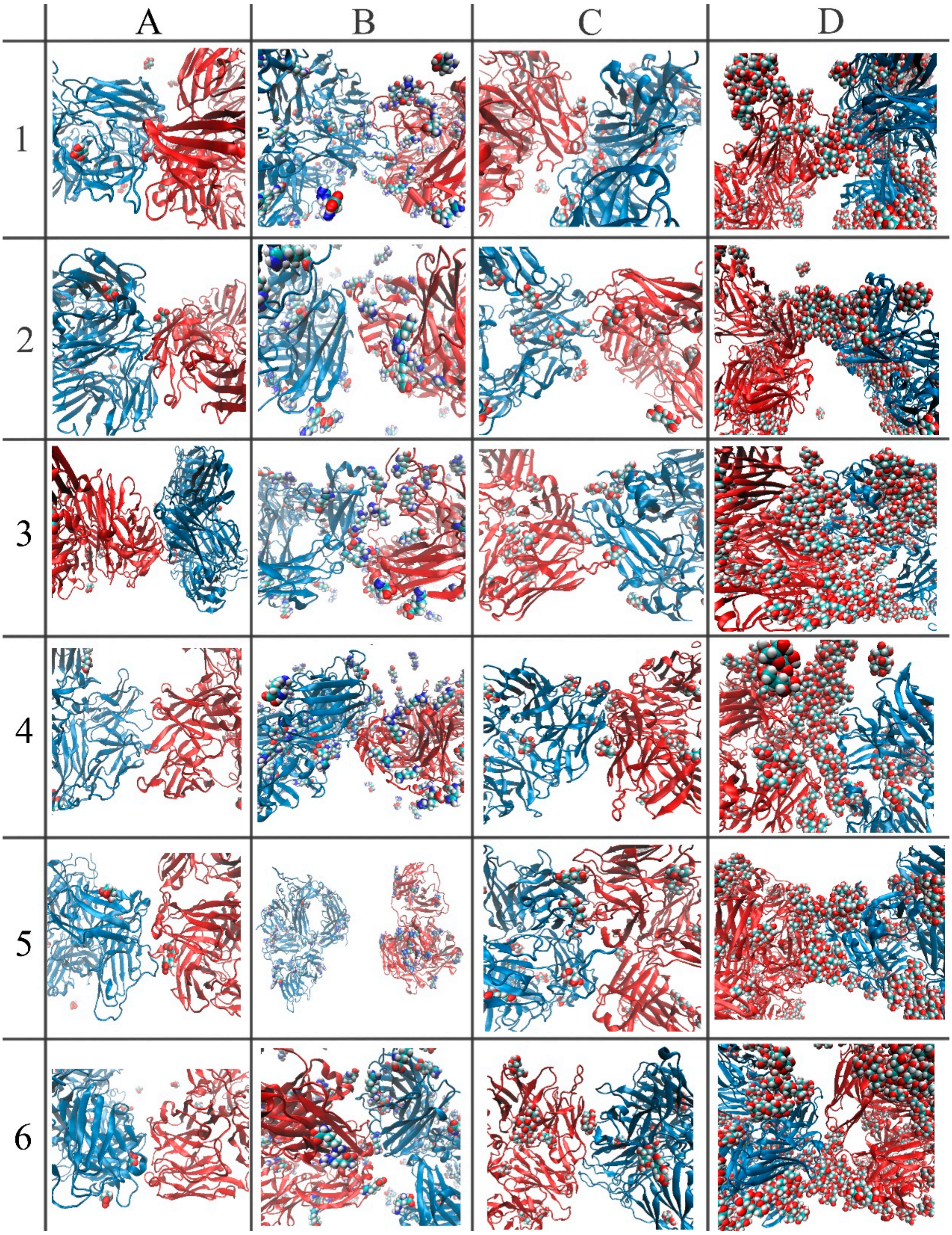

Figure S6. Aggregation structures under different initial positions and excipients conditions, where 1-6 correspond to P1-P6 respectively. (A) Succinic acid. (B) ARG+. (C) Mannitol. (D) Sucrose. All excipients were depicted using van der Waals surface.

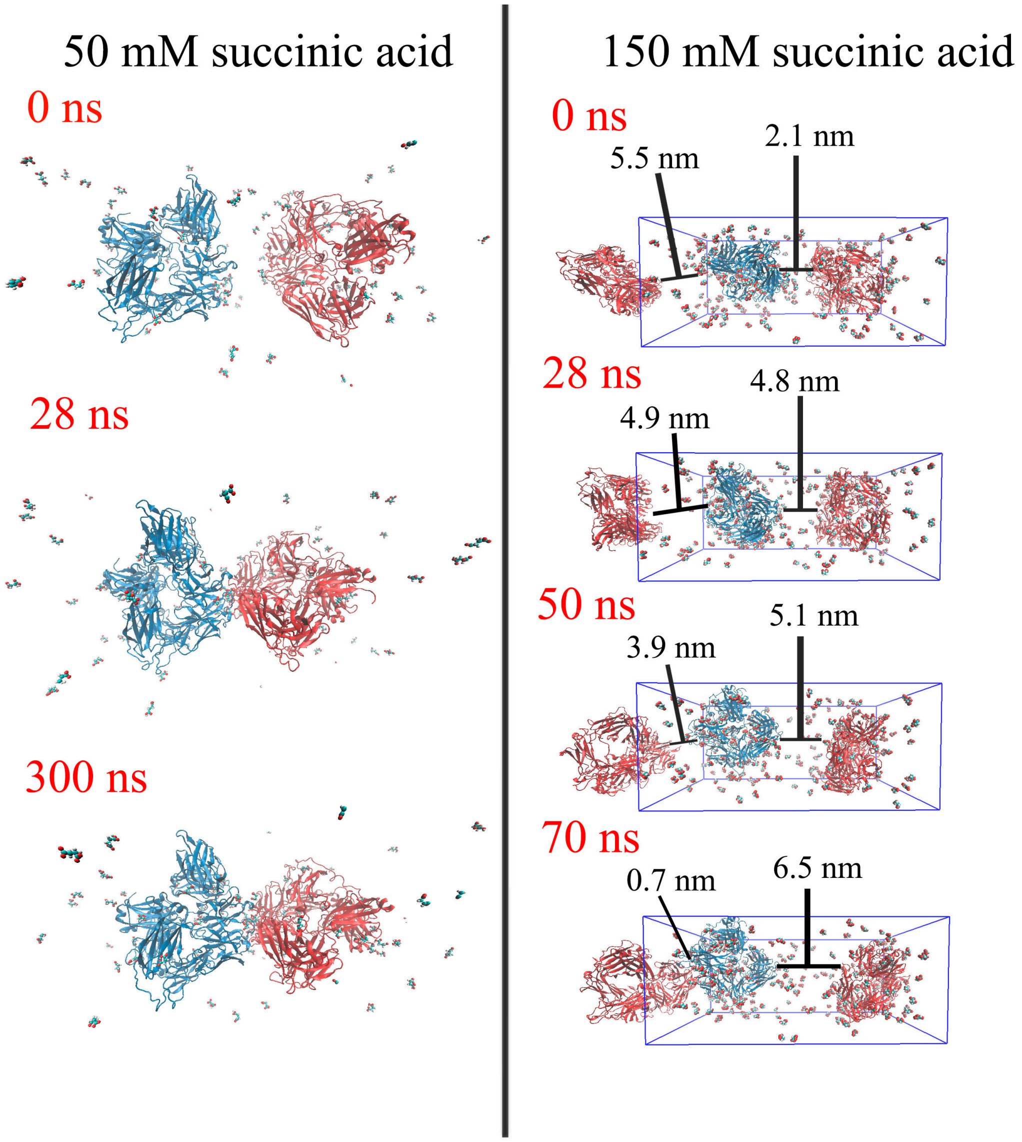

Figure S7. Aggregation trajectories of two bis-ScFvs in 50 mM and 150 mM succinic acid.

In both systems, the bis-ScFv structures initially had the same relative positions. In the 50 mM succinic acid system, the bis-ScFv molecules come closing to each other during the simulation, whereas in the 150 mM succinic acid system, the bis-ScFv molecules gradually moved apart during the simulation.

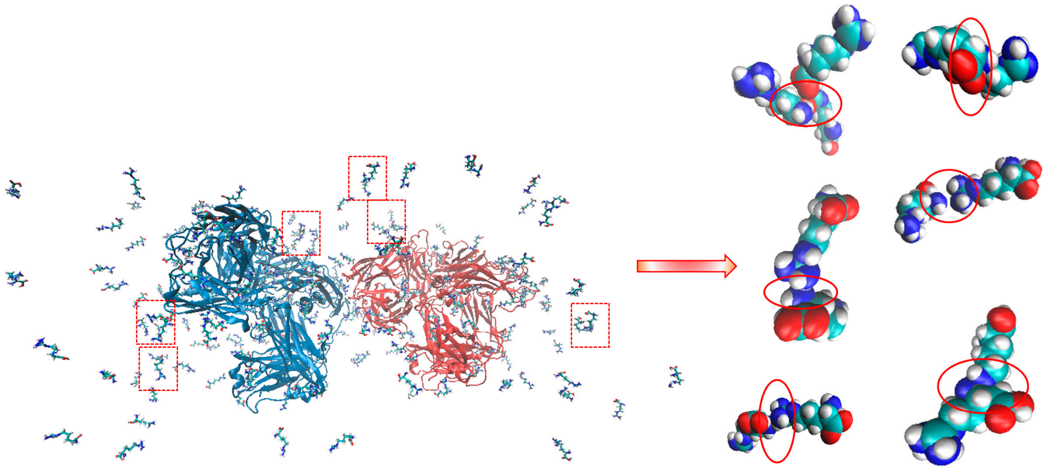

Figure S8. Self-aggregation of ARG in 150 mM ARG+ system. A significant amount of ARG+ self-aggregation was observed, with the predominant conformations being those where the aggregation occurred through interactions between -NH2 groups, both from the R group ends and the backbone.

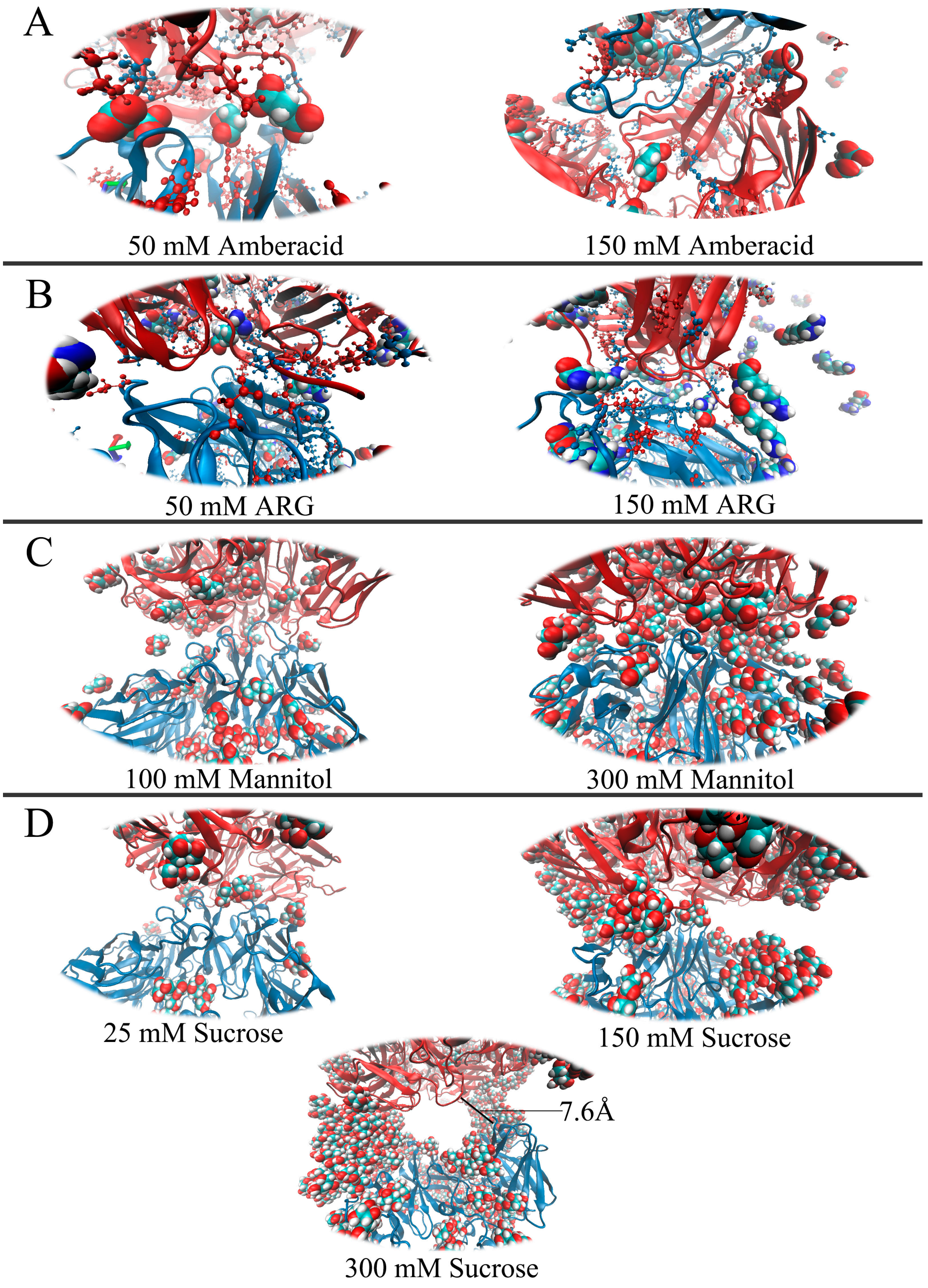

Figure S9. Contact structures between excipients and bis-ScFv at different excipient concentrations, as well as the aggregation structures of bis-ScFv.

Table S1. Modified LJ parameters of four excipients. The LJ parameters listed below are original parameters defined in the [nonbond_params] field of ffnonbonded.itp for non-water interaction calculations of excipients, rather than parameters calculated based on the Lorentz-Berthelot rule. The LJ parameters of the interaction between excipients and water remain unchanged. The LJ parameters for atom types not listed are consistent with GAFF. Succinic acid (-): oh-hydroxyl O, o-carbonyl O, hc-alkyl H. Arg (+): hn-amino H, n3-amino N, h1-alkyl H near the amino group, hc-other alkyl H, o-carbonyl O, oh-hydroxyl O. Mannitol: oh-hydroxyl O, h1-alkyl H. Sucrose: oh-hydroxyl O, h1-alkyl H, h2-H connected to furan C, os-ether O, c3-furan C. When different atomic types with the same name appear simultaneously in a .top file, the name of one of the atomic types was modified.

Excipients	Atom type	σ (nm)		ε (kJ/mol)	
		GAFF	Fitted	GAFF	Fitted
Succinic acid (-)	oh	0.307	0.330	0.880	0.920
	o	0.296	0.320	0.879	0.900
	hc	0.265	0.290	0.066	0.066
Arg (+)	hn	0.107	0.120	0.066	0.085
	n3	0.325	0.350	0.711	0.785
	h1	0.247	0.260	0.066	0.070
	hc	0.265	0.290	0.066	0.070
	o	0.296	0.320	0.879	0.900
	oh	0.307	0.330	0.880	0.920
Mannitol	oh	0.307	0.330	0.880	0.550
	h1	0.247	0.200	0.066	0.040
Sucrose	oh	0.307	0.330	0.880	0.550
	h1	0.247	0.200	0.066	0.040
	os	0.300	0.280	0.711	0.400
	h2	0.229	0.200	0.066	0.040
	c3	0.340	0.375	0.458	0.400
